# Severe Eczema Followed by Microtia Reconstruction

**DOI:** 10.1097/GOX.0000000000000462

**Published:** 2019-10-21

**Authors:** Ling Jiang, Qingguo Zhang, Yilin Cao, Yongbiao Zhang, Jintian Hu

**Affiliations:** From the * Department of Ear Reconstruction, Plastic Surgery Hospital, Chinese Academy of Medical Sciences, Beijing, People’s Republic of China; † Beijing Institute of Genomics (BIG) of Chinese Academy of Sciences; ‡ Research Center, Plastic Surgery Hospital, Chinese Academy of Medical Sciences, Beijing, People’s Republic of China

## Sir:

**Fig. 1.**
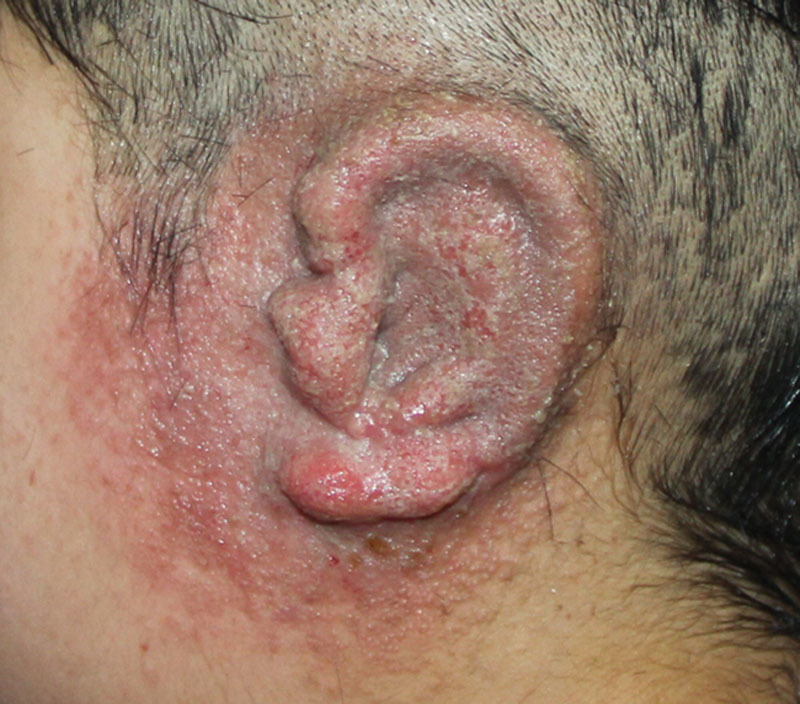
Papules appeared on and around the reconstructured auricle.

**Fig. 2.**
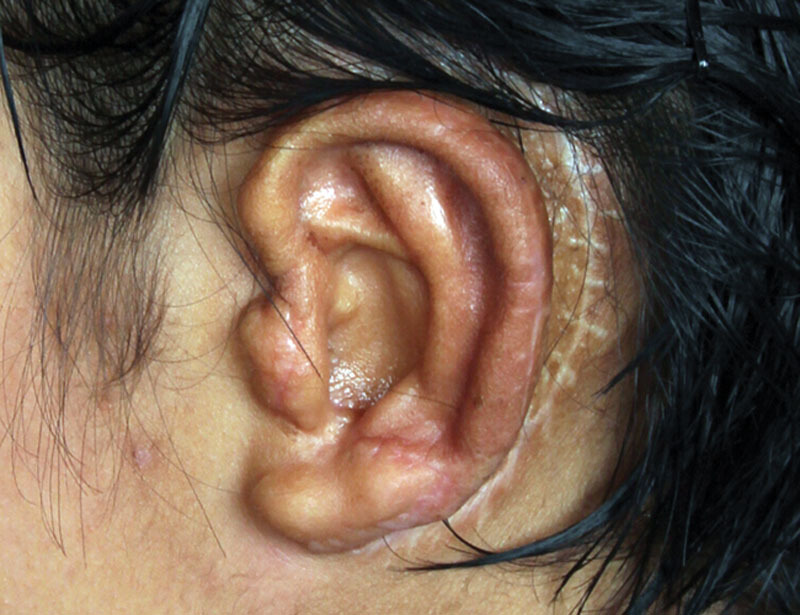
The final shape of the reconstructured auricle 12 mo after the surgery of Nagata phase 2.

In microtia reconstruction, it is typical to use the costal cartilages harvested from the patient himself or herself.^1^ After the surgery, hematoma, infection, exposed cartilage, skin, and cartilage necroses are commonly encountered.^2^ Clinically, auricle eczema is a common skin disease. In some severe cases, it is characterized by itches, erythema, papules, and herpes on and around the auricle or blister concentration with blurred edge, erosion, and scars which ooze easily. If infection worsens, pustules or pus scabs will be visible.^3^ A 9-year-old patient was admitted to ear reconstruction center for microtia repair. Nagata method was performed, the no. 7, 8, and 9 left side costal cartilages were harvested and then fixed by titanium wire. Twenty days after the surgery, itch and erythema appeared on the reconstructured auricle, and then gradually spread to the skin around it. Two days later, papules were visible on and around the reconstructured auricle and papulo-vesicle concentrated and oozed easily with blurred edge and erosion (Fig. 1). The initial treatment was normal saline wash and loratadine 5 mg qd P.O.^4^ Fourteen days later, local skin lesions around the reconstructured auricle fully recovered. Three months later when the patient showed no more symptoms of skin lesions, Nagata phase 2 was performed. Twelve months (Fig. 2) after the surgery, the recurrence of eczema was not reported in the patient follow-up.

According to “Guidelines for the diagnosis and treatment of eczema” issued by *Chinese Society of Dermatology* in 2011, the exact mechanisms of eczema are unclear.^5^ Eczema in certain area after auricle reconstruction is rare. In this case, it may be related to skin allergy caused by local iodophor disinfection after the surgery.^6^ Clinically, the skin on and around the reconstructured auricle becomes itchy, and in most cases, erythema, papules, and exudate are visible. In severe cases, it may lead to infection of the reconstructured auricle, cutaneous necrosis, and cartilage exposure. Regular treatment of eczema is usually applied, including taking antiallergic drugs, applying histamine antagonists and steroid hormone as well as local physical therapy. Clinically, it often recurs after stopping using drugs. As for prevention, it is suggested patient avoid eating or touching allergens, avoid perspiring and keep the skin clear and dry.^5^ Timely treatment are needed if abnormal itch occurs or the characteristic of the skin changes. Through timely treatment, the patient in this case is satisfied with the final shape of the reconstructured auricle. But long-term of follow-up is needed in case of recurrence.

## DISCLOSURE

The authors have no financial interest to declare in relation to the content of this article.
